# Nursing students’experience of flipped classroom combined with problem-based learning in a paediatric nursing course: a qualitative study

**DOI:** 10.1186/s12912-024-01744-z

**Published:** 2024-02-02

**Authors:** Zhi Hong Ni, Jie Huang, Dao Ping Yang, Jing Wang

**Affiliations:** Children’s Hospital of Soochow University, No. 92, Zhong nan St, 215025 Suzhou, China

**Keywords:** Nursing students, Flipped classroom, Problem-based learning, Qualitative study

## Abstract

**Background:**

Problem-based learning (PBL) is a student-centred approach that triggers learning by presenting problems cenarios early in the learning process.Flipped classrooms have been used in various disciplines using various models.Pre-class e- learning in aflipped classrooms can enrich knowledge acquisition in PBL teaching. This study was conducted to explore nursing students’experience of flipped classroom combined with problem-based learning in a paediatric nursing course.

**Method:**

This descriptive qualitative study was conducted between January and June 2022.Semi-structured interviews were conducted with nursing students who were participated in flipped classrooms combined with PBL teaching in a paediatric nursing course at Soochow university in China. Nursing students were selected using a purposive sampling method until no new data were generated (*n* = 16).

**Results:**

We identified ten sub-themes and four higher-order themes based on these sub-themes: (1)stimulating interest in learning and enhancing autonomous learning,(2)improving independent thinking and problem-solving skills,(3)cultivating team work spirit, and (4) gaining knowledge and improving skills.The findings of our research contribute to show the effectiveness of the flipped classroom combined with PBL in a paediatric nursing course.

**Conclusion:**

The flipped classroom combined with PBL in a paediatric nursing course can enhances communication and cooperation abilities among nursing students, promoting common progress and the comprehensive development of nursing students.

## Introduction

Problem-based learning (PBL) is a student-centred approach that triggers learning by presenting problems cenarios early in the learning process [1]. Barrows defined PBL as “learning that results from the process of working toward the understanding or resolution of a problem” [2], Four critical conditions for a deep approach to learning are encompassed within the PBL approach: a well-structured knowledge base, active learning, collaborative learner interaction, and a context designed to promote internal motivation through the provision of pragmatic goals (Margetson, 1994).Each student group addresses this problem in the presence of a facilitator [3].

The role of the problems cenario is to encourage students to activate their prior knowledge and stimulate their interest in the subject matter [4], thereby engaging the students in active discussions and creating a positive learning environment [5]. Several factors affect the quality of PBL scenario and its effectiveness in stimulating discussions. These factors include the reality of the scenarios, variety of experiences, degree of challenge, supporting group work, and ability to activate prior knowledge [6].PBL aims to help students define their new learning needs.

PBL is widely used in health professionals’ education [7]. where real-world scenarios provide the stimulus for a deeper understanding of both the theoretical and practical concepts in focus. Thus, PBL is a promising pedagogical model for nursing education [8]. Through PBL, students practice and develop problem-solving, self-directed learning, and collaborative skills, which are important professional skills for registered nurses. Thus, teamwork and collaborative skills are taught as part of PBL-based education.

PBL has been provento improve nursing students’ application of theory lessons in clinical practice, learning motivation, critical thinking, self-learning capabilities, and satisfaction with teaching [9–10]. As a result of recent developments in the fields of Internet technologies, social networks, and learning management systems, educators are increasingly using flipped classrooms. The flipped classroom is a blended learning methodology that combines e-learning and face-to-face classroom techniques [11]. It is intended to improve the efficacy of classroom learning by allowing students to control the timing and pace of their online learning and maximise their opportunities for active learning by engaging in class discussions and collaborative exercises in the company of peers and instructors [12]. Previous studies have demonstrated that, compared to traditional lecture-based classrooms, flipped classrooms are more effective in improving academic performance and appraisal of the course, as well as in developing engagement and higher-level thinking skills in nursing students [13–15].

Flipped classrooms have been used in various disciplines using various models. In nursing education, one of the most important teaching methods with the advantage of improving nursing students’ clinical performance and higher-level thinking abilities, PBLcan be used as an in-class activity in flipped classrooms. However, to some extent, Pre-class e- learning in a flipped classrooms can enrich knowledge acquisition in PBL teaching [16]. Few studies have investigated the combination of flipped classrooms and PBL in paediatric nursing education. [17–18].

Paediatric Nursing course is one of nursing programs’ most important core courses. The course aims to build students’ knowledge systems for caring for paediatric patients and cultivate their professional skills to assess, analyse, andsolve clinical problems. Paediatric Nursing aims to study the laws of children’s growth and development and improve children’s physical and mental health and disease care measures. Individual gender and age differences among the children are significant. Therefore, the knowledge points of paediatric disease nursing are relatively complex, and nursing students experience certain learning difficulties.Traditional in-class lectures are not conducive to the cultivation of students’ thinking abilities and are unable to promote the connection of theoretical knowledge and clinical practice skills. In view of the advantages and benefits of PBL and the flipped classroom, these methods may be suitable for changing the traditional classroom to meet the needs of the students, and a combination of these methods may produce better outcomes in the teaching of Paediatric Nursing.

The aim of this study was to explore nursing students’ experiences of applying

flipped classrooms combined with PBL in a paediatric nursing course.We conducted a flipped classroom combined with problem-based learning in a paediatric nursing course, and conducted interviews to understand students’experiences after taking the course to provide a reference for further improvement of the paediatric nursing course.

## Methods

### Design

The study used a qualitative descriptive design employing interviews [19]. The interview data were analysed using qualitative content analysis [20].Qualitative research as a systematic and subjective approach leads to increased insight, understanding and awareness of human experiences. Therefore, to discover and explain dimensions of the phenomenon in question and reveala deep understanding of the social world of the participants, an inductive qualitative research approach is most suitable.

### Participants and procedure

As previously mentioned, the purposive sampling method was used to select participants. All the participants were undergraduate nursing students in their third year who attended a flipped classroom combined with PBL in a paediatric nursing course and met the following inclusion criteria: the participants were (1) undergraduate nursing students enrolled in 2020, (2) who participated in a flipped classroom combined with a PBL paediatric nursing course, and (3) who voluntarily participated in this study. After applying the selection criteria, a total of 16 nursing students(12 female students and 10 male students) aged 20−21 years were enrolled in this study.

### Setting

This study used a qualitative design to analyse nursing students’experiences of flipped classrooms combined with PBL teaching in a paediatric nursing course. All interviews were conducted between September and December 2022 in a classroom at Soochow university.Purposive sampling was used to enrol nursing students who attended a flipped classroom combined with PBL in a paediatric nursing course.

Teaching Steps [21]:Flipped Classroom includes online pre-class teaching session and offline in-Class PBL teaching sessions.

Teachers created teaching videos, determined teaching materials, prepared teaching tasks based on course knowledge points, arranged for the students to complete learning tasks with the help of the online learning platform, and conducted PBL group in-class teaching in the PBL classroom so that the students can carry out learning activities such as discussion and sharing in the classroom.

#### Online pre-class teaching session

Each student entered the Paediatric Nursing for autonomous learning through an online course. Students must watch videos, complete homework, and take online tests independently before class. Students can participate in the platform’s interactive forum,an online communication platform for consultation if they encounter problems duringonline learning.

#### Offline in-class PBL teaching sessions

(1)Building a PBL case base.

Before the class began, teachers carefully selected and adapted real clinical cases together with medical and nursing staff, according to the requirements of the teaching objectives, to form a PBL teaching case library.

(2) Formation of PBL student groups.

Each group hadeightto ten pupils. Students with different characteristics were reasonably matched based on gender, learning capacity, personality, and other factors.

(3) Conducting PBL teaching.Students worked in small groups, each with an instructor, and were provided withcases in acts and sections. Students presented the problems to be solved in thecase scenario and discussed them in small groups to propose ways to solve the problems.

(4) Students reported, the teacher summarised, and students reported the results of the case discussions in groups. The teacher remarked on and summarised the reports from each group, briefly summarised the knowledge in correlation with the problems encountered by the students in their autonomous learning, and explained and answered questions about the students’ error-prone and confusing areas as well as common difficult problems.

### Data collection

The interviews were conducted in a quiet classroom at Soochow University. The qualitative data collection method included semi-structured, face-to-face interviews.A senior researcher (NZH) performed the interviews and trained less-experienced co-workers. NZH is an experienced Ph.D.teacher. Moreover, all researchers in this study were experienced in performing qualitative research. Initially, preliminary interviews were conducted with five students. The data from the preliminary interviews were not included in this study but were used to modify the interview structure according to the preliminary outcomes. The final interviews used in this study included the following items:

The nursing studentswere asked:(1)What are your thoughts and reflections after attending the paediatric flipped classroom combined with the PBL course?(2)What do you think is the impact on learning after attending the paediatric flipped classroom combined with PBL course?

To capture nursing students’ experiences of the paediatric flipped classroom combined with PBLcoursesin real-time,we conducted one-on-one interviews with nursing students after they completed the paediatric problem-based learning programme.Only the nursing students and interviewers were present during the interviews. No other participants were allowed to enter the interview room.Each interview lasted 30–45 min. Data were collected continuously until no new events occurred, thereby achieving data saturation [22].Audio recordings were used to collect data and field notes were taken after each interview.

### Data analysis

For the qualitative content analysis [20]the interviews were first transcribed word-by-word, and then the interview notes were compiled. Data analysis was conducted using the NVIVO software (QST International, Cambridge, MA, USA).The investigators read the transcripts to familiarise themselves with the data. Then they extracted the most relevant words and phrases to describe the nursing students’experiences in paediatric flipped classrooms combined with PBL course. The investigators read all transcripts and extracted sentences that conveyed the most meaningful information regarding the nursing students’experiences. This was followed by the preparation of coding sheets, grouping of the data, and creation and abstraction of categories. Data categorisation was performed multiple times by investigators who worked closely together until the four main categories were identified. As a confirmatory test, the four categories were shown to the caregivers, who agreed that the results accurately represented their experiences [20].

### Ethical considerations

This study was performed in compliance with the established ethical guidelines expressed in the Declaration of Helsinki.This study was approved by the Ethics Committee of the Children’s Hospital of Soochow University (approval no. #2021cs196).At the time of the study,the authors had no student–teacher relationship with involved participants. Verbal and written informed consent was obtained fromall participants before the interview took place. All participants were informed of their right to withdraw from the study at any time, without giving any reason for withdrawal. All participants were provideda guarantee of confidentiality by coding the transcripts and assigning each quotation a code.

## Results

Through data analysis, we identified the following four themes:(1)stimulating interest in learning and enhancingautonomous learning; (2)improving independent thinking and problem-solving skills; (3)cultivatinga teamwork spirit; and (4) gaining knowledge and improving skills (Table 1). Each theme is described below with supporting quotes from the participants.

**Table 1:** Themes and Sub-themes

Themes	Sub-themes
(1) Stimulate learning interest and enhance autonomous learning	Proactively learning
	Active learning atmosphere
(2) Improve independent thinking and problem-solving skills	Independently think about problems
	Positive thinking
(3) Cultivate teamwork spirit	Thinking collision and supplement missing knowledge
	Proactively and enhance communication
	Gurop collaboration and team growth
(4) Gain knowledge and improve skills	Knowledge Extension
	Improvement of cognitive skills

### Stimulate learning interest and enhance autonomous learning

#### Proactively learning

Before class, the nursing students searched for materials and discussed them with group members through the teaching content and cases posted by the teacher, thereby creating a strong learning atmosphere.

‘Before class, I try my best to understand the learning content and search for knowledge according to the tasks assigned by the teacher.Because I need to report on the class,I hope to vividly explain what I understand and learn. I will search for extended knowledge online and practice many times beforeclass.’—Participant 16.

‘Before class, the teacher assigned paediatric cases for us to think about, to answer the questions, I searched a lot of literature and actively thought’.—Participant 6.

‘I liked the combination of flipped classroom and PBL course teaching. In the course, we organised discussions and speeches; therefore, we must carefully study the relevant knowledge before class.—Participant 3.

The combination of the flipped classroom and PBL course teaching can improve students’interest in learning. During pre-class preparation and learning in the class, students are able to proactively identify problems and actively discuss and solve them.

‘I think this teaching method will encourage us to learn the paediatric course ourselves, actively collect various knowledge related to the paediatric nursing course, and make learning more proactive’.—Participant 1.

I think the flipped classroom combined with PBL course teaching is very interesting, which can better motivate students and exercise our ability to learn independently.—Participant 8.

#### Active learning atmosphere

Flipped classrooms combined with PBL course teaching adopta group teaching mode. Under the teacher’s guidance, students freely expressed their opinions. Most students expressed that an active teaching atmosphere made them more willing to participate in learning activities.

‘The teacher’s humourous and witty language, appropriate guidance, and positive responses from team members made the entire classroom atmosphere more lively. Different people have different thinking patterns, and their emphasis on answering questions varies. Therefore, I have a more comprehensive understanding of the learning pointsafter class’.—Participant 12.

‘We gathered with the teachertogether to discuss the case, which brought us closer to the teacher. The entire format and atmosphere are also relatively relaxed; just like a few friends gather to discuss and learn, the teacher can have a deeper understanding of each student.—Participant 15.

### Improve independent thinking and problem-solving skills

#### Independently think about problems

A flipped classroom combined with PBL course teaching is no longer a teacher’s lecture but student-centred teaching, which can enable students to develop the habit of independent thinking and analysis of problems.

‘Discovering their strengths and weaknesses while reported from other groups is also an improvement for oneself’.—Participant 2.

‘We will preview before class based on the learning materialsreleased by the teacher, and check the materials for any questions we cannot understand’.—Participant 5.

‘In group discussions and speeches, everyone has their own opinions, which has helped me develop the habit of diligent thinking’.—Participant 11.

‘Combining the case study and analysis provided by the teacher expanded our thinking’.—Participant 7.

Students believe that it is very important to be able to think about problems actively rather than passively listening to teachers’ indoctrination when combining PBL teaching with a flipped classroom.

‘In the paediatric nursing course learning,it gave me the feeling I had before, which was to think proactively’.—Participant 4.

‘During the problem discussion section,the teacher asked a question, and she asked us to review the materials ourselves and think for ourselves. We would check the materials ourselves in and out of class, which would feel better.—Participant 14.

#### Positive thinking

Most students realised that teachers are not simply imparting knowledge but rather combining practical cases and specific clinical nursing problems, asking students questions, and guiding them to find answers, thereby triggering positive thinking among students.

‘In PBL class, my attention is very focused. We will identify problems together and then thinkabout and discuss them with classmates. Compared with regular classes, this can improve thinking ability.—Participant 11.

‘When we learned the PBL course about children’s respiratory disease and bronchopneumonia, the teacher asked us how to observe the changes of hypoxia in children with pneumonia according to the on-site case study. We all expressed our own opinions, and the teacher also encouraged us to share our ideas so that we could have our own ideas.—Participant 9.

#### Actively ask questions and deepen understanding

Students proposed that, compared with traditional teaching, flipped classrooms combined with PBL curriculum teaching are more willing to ask questions and understand what they do not understand in the course of classroom discussion.

‘In PBL class, everyone can answer questions and solve problems with each other. If there are any problems, they can be solved through group discussions. If they cannot be solved within the group, they can work together with the teacher or with the entire class to solve them. This is very good.’—Participant 10.

During normal classes, it is necessary to follow teachers’ ideas, and areas that are not understood are easily overlooked.

‘Because PBL discussions require everyone in the group to raise questions, it is necessary to read thepaediatric nursing textbooks and identify the areas of your doubts’.—Participant 13.

‘I can learn from this classroom feedback what I need to strengthen and do. I won’t feel defeated; just treat it as a constructive thing.—Participant 16.

PBL coursescan broaden students’knowledge horizontally and deepen their understanding of disease knowledge vertically. Most students believe that PBL course methods are novel and diverseand can stimulate interest and deepen their understanding.

‘For example, there is PBL teaching for children with congenital heart disease. The teacher will notify us in advance and let us find pictures of congenital heart disease, such as cardiac vascular anatomy, angiography, etc., and share them in class. This teachingmethod can help us understand the formation of congenital heart disease and other knowledge.—Participant 2.

‘I feel that after attending the PBL course, I have gained a deeper understanding of paediatricdiseases, and a clearer understanding and observation of clinical manifestations’.—Participant 6.

### Cultivate teamwork spirit

#### Thinking collision and supplement missing knowledge

During the PBL discussion process, it is possible to identify and fill in gaps, supplement overlooked knowledge, and comprehensively grasp the course content.

‘Sometimes, I may not notice the questions raised by other classmates, so I tend to pay more attention in the study. Sometimes, I feel that other classmates’ideas are not correct, and I also bring them up to them.’—Participant 4.

‘During the discussion, if some students have different opinions, they will also raise them, and everyone will have a collision of ideas so that the answer will be more comprehensive’.—Participant 15.

#### Proactively and enhance communication

Through PBL discussions, students can get along harmoniously and better understand each other.

‘When we discussed the question, we raised the question and then solved the question, which leads to dialogue and gradually becomes familiar with them’.—Participant 7.

‘I tend to be distracted in my study. There will be a question-answering session in the flipped classroom that will improve my interest in learning. It’s very good’.—Participant 9.

#### Gurop collaboration and team growth

In a flipped classroom that combines PBL course learning, team members conduct preclass previews according to a course planorganised by the team leader. This requires independent access to relevant materials, problem-solving, and, more importantly, a reasonable division of labour among team members to share learning methods with each other.

‘Classmates study together to divide and collaborate, check materials, write reports, etc., and then several classmates discuss learning outcomes together, which is better than learning alone’.—Participant 3.

‘We work together as a group of classmates, each of whom has their strengths and can learn from them to make up for my weaknesses. As a member of the group, I must also conscientiously complete the relevant content.—Participant 1.

‘Although we have all assigned learning tasks, each stage requires everyone to discuss and learn together, communicate with each other, and reach an agreement’.—Participant 12.

When I encounter problems that I don’t understand, I will consult with my group members and actively learn to improve my learning outcomes.’—Participant 14.

In the class, team members collaborate and answer questions in an interactive manner. This shared learning method can promote the overall growth of teams. They realised that listening to the opinions of other team members can help them achieve alternative ways of thinking and enhance their thinking processes.

‘By adopting this teaching method, we can have a more comprehensive understanding of our own shortcomings and blind knowledge spots, which can help us think more comprehensively and effectively’.—Participant 8.

‘The biggest difference between flipped classroom combined with PBL teaching and regular teaching is that it requires our active cooperation. From the preparation of materials, we need to combine the outline and case studies to review relevant materials and actively communicate with other students so that everyone can work together to reach the best conclusion.—Participant 13.

‘Engaging in self-learning, discussion, and raising one’s own questions or opinions on a certain issue among group participants can not only make group learning more effective but also promote everyone to develop good learning habits’.—Participant 15.

### Gain knowledge and improve skills

#### Knowledge extension

The flipped classroom combines PBL course teaching, which encourages students to find diversified teaching resources and prepare fully before class. During the teaching process, the teacher helps students comprehensively learn the course from multiple perspectives, such as theoretical knowledge, clinical applications, and patient needs, thereby helping them expand their knowledge.

The teacher also guided us on the concept of neonatal developmental care, which is not mentioned in our paediatric nursing book but has important clinical significance. The flipped classroom combined with the PBL course not only allows us to focus on books but also broadens our horizons..’—Participant 14.

‘Flipped classrooms that combine PBL courses with paediatric cases usually involve knowledge from multiple disciplines. By discussing and learning about this case, we can not only learn the knowledge within the textbook but also expand it to other knowledge outside the textbook, which helps us integrate knowledge from various disciplines’.—Participant 3.

#### Improvement of cognitive skills

Flipped classrooms combined with PBL teaching can shorten the process of disease knowledge development from theoretical learning to practical applications. Some students mentioned that disease knowledge and nursing measures learned in flipped classrooms combined PBL courses could help them achieve better results in clinical health education and nursing practice.

‘By studying cases of various paediatric diseases, we can connect knowledge with practical applications, so that we can directly apply the medical knowledge we have learned to clinical cases’.—Participant 12.

‘Through flipped classroom combined with PBL course teaching, I think that when nursing patients with such diseases, I can find their nursing problems quickly, which is more targeted. Patients also think that I am a professional.—Participant 2.

Most students have a strong sense of benefit from the combination of flipped classrooms and PBL teaching and can gain useful knowledge and experiences from the curriculum.

‘For the flipped classroom teaching combined with PBL course teaching, I think I have mastered a lot of knowledge in the classroom. During the internship, when I performed a physical examination for children, I felt that my professional knowledge was very comprehensive and more confident. I can answer all the questions asked by the children’s families.—Participant 5.

‘I think that compared with ordinary teaching, Flipped classroom combined with PBL course teaching course is very helpful to me. This enables me to better understand the clinical manifestations, nursing problems, and nursing measures of paediatric diseases.—Participant 8.

## Discussion

This study explored nursing students’experiences of flipped classrooms combined with PBL in a paediatric nursing course and found that nursing students stimulated learning interest and enhanced autonomous learning,improved independent thinking and problem-solving skills,cultivated teamwork spirit, and gained knowledge and improved skills after attending a flipped classroom combined with PBL in a paediatric nursing course [23–24].

Compared to the traditional teaching mode, through a flipped classroom combined with PBL course learning, students can complete learning within a certain range according to their own schedule, freely choose learning places, and ensure that they can complete the course in a better state [25]. Hanet al proposed that in a flipped classroom, improving the learning effect is conducive for students who are good at managing time [26]. Therefore, to ensure the effectiveness of students’self-directed learning, teachers must strengthen the supervision and guidance of students’ self-directed learning and implement personalised self-directed learning.

The traditional teaching model advocates the leading role of teachers, neglecting students’initiative and creativity [13]. Before entering the flipped classroom,mastery of the content increases students’ intuitive learning skills and confidence to perform classroom activities [27]. Studies have also shown that flipped classrooms improve students’ self-directed learning readiness and increase their satisfaction [28]. There is evidence suggesting that the flipped classroom approach positively affects students’ motivation and significantly improves self directed readiness skills [29–30].The flipped classroom teaching model combined with PBL can develop nursing students’ interest in learning and meet learners’ needs. nursing students can choose the most proper ways to obtain new knowledge [31].

This study transforms the role of teachers into that of initiators and coordinators of the curriculum and transforms teacher-centred education into putting students at the centre creates an environment and atmosphere for active learning.

Mortensen and Nicholson [32]proposed that active learning could promote students’independent thinking and increase their creativity. The results of this study showed that the flipped classroom teaching model combined with PBL improved students’ learning outcomes to some extent by actively discussing issues and reducing classroom-learning loopholes.

In this study, students raised questions in classroom discussions and engaged in brainstorming through group discussions, which was beneficial for promoting mutual learning and progress among students as well as enhancing communication between teachers and classmates. Research has shown that group discussions can help students experience a spirit of teamwork and cultivate team qualities [33–34]. Schlairetetal [35]. proposed that flipped classrooms allow teachers sufficient time to observe students’learning and provide targeted guidance. Therefore, teachers, in addition to preparing well before class, should not ignore effective guidance for students.

The flipped classroom combines PBL course-teaching methods to develop students’skills in various areas, such as problem-solving, time management, teamwork, higher-level thinking, and the ability to obtain and evaluate information and use it to construct knowledge flexibly [36–37].However, a drawback of the PBL teaching method is the lack of systematically acquired information [38–39], and the lack of effective teaching resources can significantly affect PBL teaching. This study used an online course platform to fully meet students’self-study needs before and after classes. It was combined with offline PBL teaching to fully integrate the optimal online and offline teaching resources for the flipped classroom model, which improved students’ problem-solving and clinical thinking abilities while promoting the systematisation and integrity of knowledge acquisition andachieving good teaching results.

The flipped classroom teaching model combined with PBL was recognised by the students, and the traditional teaching model based on a teacher’s lecture could give full play to the teacher’s organizational role in classroom teaching. However, it ignores students’ initiative,creativity, and cognitive subject roles, which is not conducive to improving their interest in learning and autonomous learning abilities. Furthermore, there is insufficient teacher-student and student-student communication, and the classroom atmosphere is not active in the traditional teaching model [40].

The flipped classroom combined with PBL not only promotes students’ autonomous learning of basic concepts and knowledge beforehand by using online resources, but, especially in the process of finding answers andsolving problems based on PBL cases, students also need to retrieve andlearn relevant knowledge independently. This is conducive to promoting students’ subjective initiatives and improving their autonomous learning ability, in line with the results of Zeng andZhao [41–42].

As an important core educational course in the nursing profession, paediatric nursing not only emphasises the mastery of basic knowledge and concepts but also focuses on the cultivation of students’ clinical thinking and practical problem-solving abilities. Based on real clinicalsituations, the flipped classroom combined with PBL encourages students to find, analyse, and solve problems in the process of independent exploration, which isconducive to promoting the cultivation of students’ clinical thinking ability and shortening the distance between school and the clinic [43]. We found that students can obtain a greatsense of self-satisfaction in the process of independent analysis and problem-solving, whichis beneficial for promoting their self-improvement and increasing their interest inlearning. Moreover, the university stage is a key period of life development, and having good communication and cooperation skills is the basis for nursing students to improve their comprehensive quality, clinical nurse-patient communication skills, and health careteam cooperation skills [44].

### Strengths and limitations

This study has both strengths and limitations. One strength is that the setting and the different stages of the research process, namely,data collection and analysis, were clearly described so that readers could follow the process, enhancing the credibility and transferability of this study. Quotations directly lifted from the interviews were included in the results section to illustrate that each category originated from the student’s perspective, thus creating transparency in the content analysis.

This study used the purposive sampling method, which can limit the transferability of the results. However,researchers attempted to use maximum variation by considering the participants’ characteristics regarding age,gender, and a proportional number of participants for qualitative studies were used.

## Conclusion

The flipped classroom combined with PBL in a paediatric nursing course can stimulate learning interest, enhance autonomous learning,improve independent thinking and problem-solving skills,cultivate teamwork spirit, gain knowledgeand improve skills. Which is beneficial for promoting nursing students’ self-improvement and increasing their interest inlearning.

## Data Availability

The data that support the findings of this study are available from the corresponding author upon reasonable request.
